# Healthcare at Risk: Why Do Sudan’s Healthcare Workers Face Gaps in Hepatitis B Virus Protection?

**DOI:** 10.7759/cureus.79745

**Published:** 2025-02-27

**Authors:** Mohamed A Abdelrahim, Mahil Abdalla, Eram Yousif, Ragda Abdallah, Abdelhadi A Elsayed

**Affiliations:** 1 Anatomical Sciences, St. George's University School of Medicine, St. George, GRD; 2 Physiology, Neuroscience, and Behavioral Sciences, St. George's University School of Medicine, St. George, GRD

**Keywords:** awareness gaps, exposure risk, hcws, healthcare workers, hepatitis b, immunization, needlestick injuries, sudan, sudan khartoum, vaccination

## Abstract

Background

Hepatitis B, a widespread and potentially life-threatening viral infection, affects millions globally. Transmission occurs through exposure to the bodily fluids of infected individuals, placing healthcare workers (HCWs) at heightened risk due to frequent contact with patients. This study evaluated hepatitis B vaccination rates, causes of incomplete immunization, and incidents of needlestick injuries among HCWs at Khartoum Teaching Hospital, Sudan.

Methods

Seventy-three HCWs from the different departments of Khartoum teaching hospital departments were recruited. Participants completed a self-administered questionnaire following informed verbal consent. The collected data focused on hepatitis B vaccine adherence and barriers to full vaccination.

Results

Of the 73 HCWs (primarily aged 20-30 years), 30 (41.1%) had completed the three-dose vaccination regimen as per guidelines. In contrast, 14 (19.2%) received partial doses, and 29 (39.7%) were unvaccinated. The predominant reason cited for incomplete or absent vaccination was vaccine unavailability. Additionally, 25 (34.2%) reported prior needlestick injuries or exposure to contaminated materials, with fully vaccinated HCWs showing a statistically significant association with reduced exposure risk (p<0.05).

Conclusion

Inadequate vaccine coverage and supply shortages resulted in incomplete immunization among HCWs. Contributing factors include insufficient awareness and underestimation of hepatitis B’s severe outcomes by medical professionals.

## Introduction

Hepatitis refers to liver inflammation. It can be caused by a variety of different viruses, including the hepatitis B virus (HBV) [1]. HBV is among the most prevalent and severe liver infections globally, impacting millions [2]. Acute cases may present with jaundice or elevated serum aminotransferase levels. Chronic HBV infections can remain asymptomatic or progress to liver cirrhosis or cancer [3]. Transmission occurs through contact with the bodily fluids of infected individuals [1]. Healthcare workers (HCWs) routinely face exposure risks while caring for patients with hepatitis B infection [4].

According to the WHO, HBV chronically infects approximately 254 million (12.7.%) [5] of the over two billion people exposed globally [5]. Annually, about four million acute HBV cases occur, resulting in one million deaths due to chronic complications [2]. The Centers for Disease Control and Prevention (CDC) estimated that approximately 12,000 HCWs in the U.S. become infected with HBV each year due to occupational exposure. Of those affected, 500 to 600 require hospitalization due to the severity of their infection. Additionally, 700 to 1,200 develop chronic HBV, increasing their risk of serious complications such as cirrhosis and liver cancer [4]. High-endemic regions (≥8% prevalence) include sub-Saharan Africa, Southeast Asia, and parts of the Middle East [6]. While the exact prevalence of HBV among HCWs in Sudan remains uncertain, it is estimated to be comparable to that of the general population, which is more than 8% [6].

While HBV is vaccine-preventable, global eradication has not been attained yet due to persistent carrier populations and ongoing disease burden, necessitating continued therapeutic efforts [2,5]. Vaccines are composed of an HBV surface antigen (HBsAg), produced via plasma-derived or recombinant DNA methods. The standard HBV schedule includes three doses at zero, one, and six months, providing antibody protection in over 95% of infants, children, and adolescents, and over 90% of adults [5]. The World Health Organization (WHO) advocates universal inclusion of HBV vaccines in national immunization programs [2].

Despite accessible vaccination protocols, HCWs remain vulnerable. Studies on HCW vaccination rates yield inconsistent findings, highlighting the need for precise data on immunization adherence and barriers. The study aimed to evaluate hepatitis B vaccination adherence among HCWs in Sudan by assessing the proportion of fully immunized individuals and identifying barriers to incomplete vaccination. Additionally, it evaluated booster dose uptake, awareness of antibody titer testing, and quantified occupational exposure to needlestick injuries to comprehensively address gaps in immunization practices and occupational safety.

## Materials and methods

Study design

This study utilized a cross-sectional analytical design to evaluate HB vaccination patterns among HCWs at Khartoum Teaching Hospital. The cross-sectional methodology was selected to capture data at a single time point, allowing for an efficient assessment of relationships between key variables such as vaccine uptake and occupational exposure. This approach was particularly suited to the study’s resource-limited setting, balancing logistical feasibility with the need to analyze critical associations.

Study setting

The research was conducted at Khartoum Teaching Hospital, situated 2 kilometers from the center of Sudan’s capital. As the largest tertiary care facility in Khartoum, the hospital serves a diverse patient population and operates as a national referral center for complex medical cases. Its services span multiple specialties, including surgery, internal medicine, obstetrics and gynecology, orthopedics, and laboratory medicine. Additionally, the hospital functions as a training site for medical students and residents affiliated with the University of Khartoum’s Faculty of Medicine. The selection of this site was driven by its prominence as a national healthcare leader, its large and heterogeneous workforce, and its representativeness of low-resource settings, ensuring findings could be generalized to comparable contexts.

Study population

Inclusion and Exclusion Criteria

The study population included all full-time and part-time HCWs employed at the hospital, encompassing nurses, house officers, medical officers, registrars, consultants, laboratory specialists, and technicians. Exclusion criteria were applied to non-HCW staff, such as administrative personnel and janitorial workers, as well as individuals not formally employed by the hospital, including volunteers and visiting practitioners. A convenience sampling approach was adopted to accommodate logistical challenges and the need for rapid data collection in a high-volume clinical environment. While this method may introduce selection bias, it prioritized practicality given the operational constraints of the setting.

Sample Size Calculation

The sample size was determined using the standard formula for cross-sectional studies, $n=\frac{{Z}^{2}P\left(1-P\right)}{{d}^{2}}$, where n is the sample size, Z is equal to 1.96 (reflecting a 95% confidence level), P is 0.045 (the estimated prevalence of incomplete vaccination from prior literature [1]), and d=0.05 (a 5% margin of error). This calculation yielded a minimum sample size of 66 participants. To account for potential non-response, the target was adjusted upward by 10%, resulting in a final sample of 73 participants. The total population of eligible HCWs was considered to ensure representativeness. While a convenience sampling approach was employed to accommodate the hospital’s demanding clinical environment, efforts were made to include a broad range of HCWs to strengthen the study’s representativeness.

Data collection and analysis

Data were gathered using a structured, pre-tested questionnaire. The instrument included both closed- and open-ended questions and underwent preliminary testing with eight HCWs (excluded from the final sample) to refine clarity, relevance, and response options. Revisions included simplifying technical terminology and adding illustrative examples to open-ended prompts, such as requesting participants to “Describe reasons for not completing vaccination.” Variables assessed encompassed demographics (e.g., age, gender, specialty, department, years of service), vaccination status (including doses received, completion rates, sources of vaccination), and occupational risks (e.g., history of needle-stick injuries and reporting practices). Participants self-administered paper-based questionnaires during their shifts over a four-week period in July 2012 to maximize engagement. Anonymized coding ensured participant confidentiality throughout the process.

Data were analyzed using IBM SPSS Statistics for Windows, Version 16.0 (Released 2007; IBM Corp., Armonk, NY, United States) through a two-stage approach. Descriptive statistics summarized categorical variables (e.g., vaccination rates) as frequencies and percentages, while continuous variables (e.g., age) were reported as means. Bivariate analysis employed chi-square tests to explore associations between variables and vaccine completion, with statistical significance set at α=0.05.

Ethical considerations

Ethical approval was obtained from the Ethical Committee of the University of Khartoum’s Faculty of Medicine. Informed consent was obtained from all participating HCWs. Each participant received a thorough explanation of the study’s objectives, procedures, benefits, and risks to ensure that all requirements for informed consent were fully met. This verbal consent process was reviewed and approved by the ethical committee. We have taken steps to safeguard HCWs' privacy and ensure anonymity. Data anonymization protocols were strictly followed, with identifiers stored separately and accessible only to the principal investigator. Participants retained the right to withdraw from the study without consequences.

## Results

Participant demographics and occupational characteristics

A total of 73 HCWs from Khartoum Teaching Hospital participated in this study. The cohort comprised a slightly higher proportion of males (38, 52.1%) compared to females (35, 47.9%). Age distribution skewed toward younger participants: 51 (69.9%) were aged 20-30 years, 17 (23.3%) were 31-40 years, and only five (6.8%) were over 40 years. Occupational roles varied, with house officers representing the largest subgroup (28, 38.4%), followed by nurses (20, 27.4%) and registrars (13, 17.8%). Smaller proportions included medical officers, laboratory specialists, and technicians (12, 16.4%) (Table 1).

**Table 1:** Professional roles of the HCWs in the hospital. HCWs: healthcare workers

	Frequency	Percent
House officers	28	38.4
Nurses	20	27.4
Registrars	13	17.8
Medical officers	5	6.8
Lab specialists	5	6.8
Lab technicians	2	2.7
Total	73	100

Most participants had limited work experience: 52 (71.2%) had been employed for less than five years, 14 (19.2%) for five to 10 years, and seven (9.6%) for over 10 years. Primary workspaces included hospital wards (29, 39.7%), emergency rooms (15, 20.5%), and combined ward/ER settings (20, 27.4%). Smaller groups worked in laboratories (seven, 9.6%) and Intensive Care Units (two, 2.7%) (Table 2).

**Table 2:** Departmental distribution of the HCWs in the hospital.

	Frequency	Percent
Ward	29	39.7
Emergency room & ward	20	27.4
Emergency room	15	20.5
Laboratory	7	9.6
Intensive care unit	2	2.7
Total	73	100

Vaccination coverage and determinants

**Figure 1:**
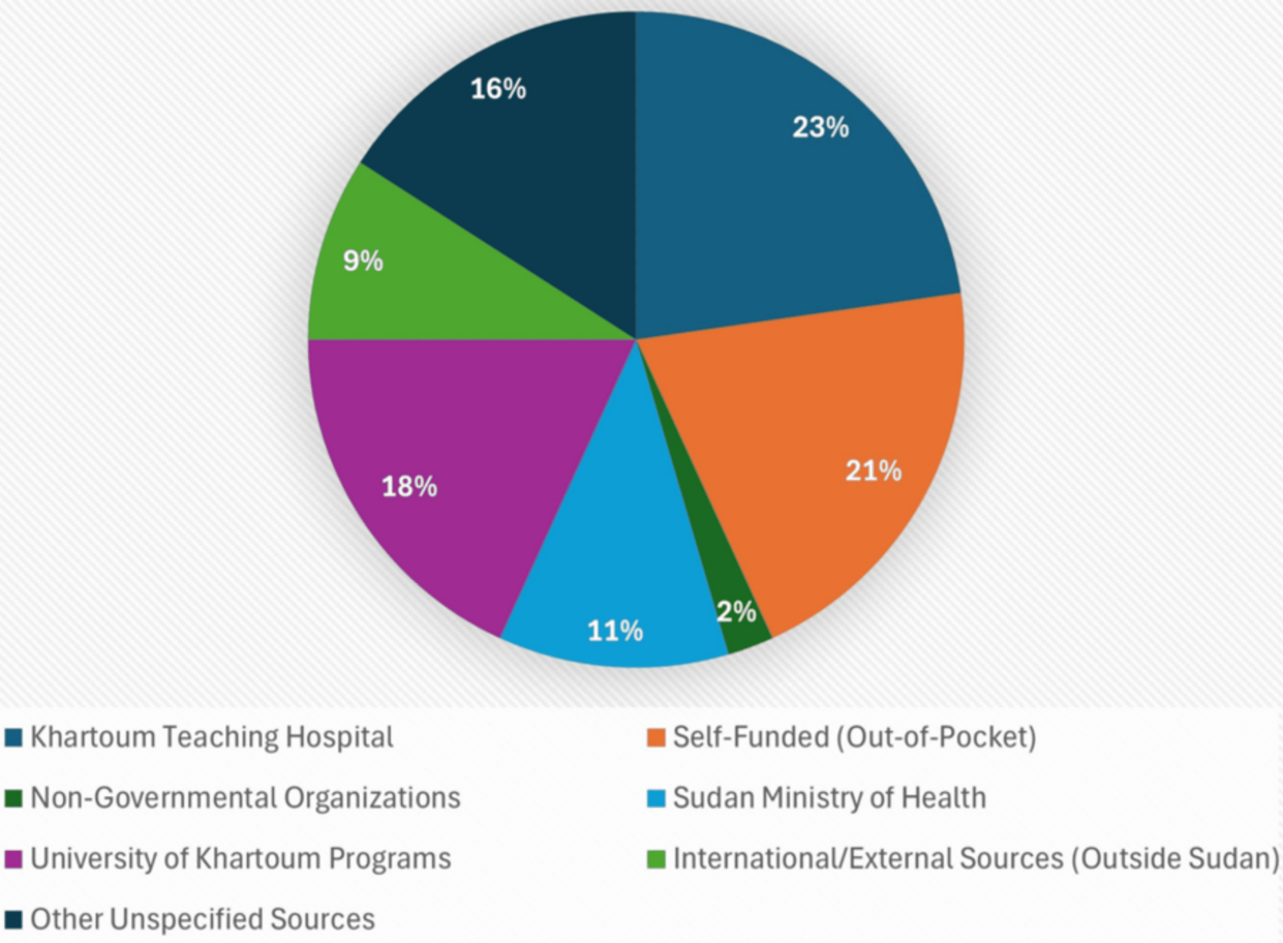
Sources of hepatitis B vaccination among the HCWs in the hospital “n = 44 (60.3%)”. HCWs: healthcare workers

Hepatitis B vaccination coverage was suboptimal. Only 30 HCWs (41.1%) had completed the full three-dose schedule, while 29 (39.7%) had not received any doses, and 14 (19.2%) reported incomplete courses. Among the 44 vaccinated individuals, vaccine acquisition pathways included Khartoum Teaching Hospital (10, 22.7%), out-of-pocket payments (nine, 20.5%), and University of Khartoum programs (eight, 18.2%). Smaller proportions sourced doses from the Sudan Ministry of Health (five, 11.4%), non-governmental organizations (one, 2.3%), or international/external providers outside Sudan (four, 9.1%) (Figure 1).

The predominant barrier to vaccination was vaccine unavailability, reported by 27 (61.4%) of unvaccinated or partially vaccinated participants. Additional reasons included high cost (seven, 15.9%), negligence (three, 6.8%), time constraints (two, 4.5%), and unreported causes (five, 11.4%). Among the 44 previously vaccinated HCWs, only one (2.3%) had received a booster dose, highlighting low booster uptake. Only four (5.5%) had ever undergone antibody titer testing to confirm immunity, indicating gaps in long-term immunity monitoring.

Occupational exposure and vaccination association

The statistical analysis of the relationship between HCWs' characteristics and their vaccination status revealed no significant associations for most factors. A Chi-square test was conducted for categorical variables, and p-values were reported to determine statistical significance. Gender (p = 0.52), age group (p = 0.40), specialty (p = 0.56), duration of work experience (p = 0.32), receipt of a booster dose (p = 0.41), and presence of antibody titers (p = 0.19) showed no statistically significant association with vaccination completion. However, needlestick injury history demonstrated a significant association with vaccination status (p = 0.05), suggesting that HCWs with a history of needlestick injuries were more likely to complete their vaccination regimen (Table 3).

**Table 3:** Relationship between healthcare workers’ characteristics and vaccination status (chi-square test) (n=73). * include those not received the vaccine or not completed the required course.

Characteristics		Vaccination complete		Vaccination not complete*		Total		P-value
		No.	%	No.	%	No.	%	
Gender	Male	16	42.1	22	57.9	38	100	0.52
	Female	14	40	21	60	35	100	
Age	≤30	20	39.2	31	60.8	51	100	0.40
	>30	10	45.5	12	54.5	22	100	
Specialty	Nurses	8	40	12	60	20	100	0.56
	Doctors	22	41.5	31	58.5	53	100	
Duration	<5	20	38.5	32	61.5	52	100	0.32
	>5	10	47.6	11	52.4	21	100	
Booster	Yes	1	100	0	0	1	100	0.41
	No	29	40.3	43	59.7	72	100	
Antibody titer	Yes	3	75	1	25	4	100	0.19
	No	27	39.1	42	60.9	69	100	
Needlestick injury	Yes	14	56	11	44	25	100	0.05
	No	16	33.3	32	66.7	48	100	

## Discussion

The findings of this study reveal critical insights into the HBV vaccination status among HCWs at Khartoum Teaching Hospital, Sudan. The results highlight significant gaps in vaccination coverage, barriers to vaccine uptake, and the impact of occupational exposure on vaccination behavior. These findings are consistent with previous studies conducted in similar low-resource settings. Yet, they also underscore unique challenges faced by HCWs in Sudan, particularly in the context of ongoing healthcare system disruptions.

Vaccination coverage and regional comparisons

Only 41.1% of HCWs in our study completed the full HBV vaccine schedule, while 39.7% remained unvaccinated - a trend consistent with findings from Omdurman and Khartoum Teaching Hospitals, where 41% of HCWs had partial or complete vaccination, but only 26.19% completed all three doses [6,7]. These rates are marginally higher than those reported in Nigeria (36.2% full vaccination) [8] but remain far below high-income countries like the USA (75%) and France (93%) [9,10]. The disparity reflects systemic challenges in low-resource settings, including vaccine unavailability (cited by 64.3% of unvaccinated HCWs in our study) and reliance on out-of-pocket payments (21.4%). Similar barriers were observed in Ethiopia, where 58.2% of HCWs cited vaccine unavailability [11]. These findings collectively emphasize the urgent need for institutional vaccination programs and subsidies to alleviate financial burdens on HCWs.

Gender disparities and profession-specific trends

Notably, our study found higher vaccination completion rates among male HCWs, contrasting with Gadour et al., who reported higher uptake among females in Sudan [7]. The latter attributed this to women’s heightened awareness of HBV transmission risks to offspring, a motivation less emphasized in our cohort. This discrepancy may reflect cultural or contextual differences, such as workplace accessibility or gender-specific healthcare engagement, warranting further qualitative investigation. Profession-specific disparities were also evident: doctors had higher full vaccination rates (41.5%) than nurses (40%), consistent with a pan-African study where 52.4% of doctors versus 26.3% of nurses were fully vaccinated [12]. The lower rates in our study may reflect Sudan’s strained healthcare infrastructure, exacerbated by recent conflicts that disrupted vaccine distribution and institutional support [13].

Occupational exposure and vaccination behavior

Our study underscores a critical association between hepatitis B vaccination completion and prior needle-stick injuries, highlighting the interplay of knowledge, practice, and occupational risk perception in driving vaccine uptake. This finding aligns with studies by Siddig et al. and Gadour et al., which emphasized that higher Knowledge, Attitude, and Practice (KAP) scores correlate with improved vaccination adherence among HCWs in Sudan [6,7]. Specifically, HCWs with occupational exposure were more likely to complete the vaccine schedule suggesting that firsthand experience with occupational hazards reinforces preventive behaviors. This aligns with Siddig et al.’s observation that vaccinated HCWs had significantly higher KAP scores than unvaccinated peers [6], underscoring the role of education and risk awareness in mitigating HBV transmission.

Implications for policy and practice

The low uptake of booster doses (1.4%) and antibody titer testing (5.5%) observed in our study highlights significant gaps in post-vaccination monitoring, which is a critical component of ensuring long-term immunity. In high-income countries such as the United States, HCWs are required to undergo routine antibody titer assessments [10], a practice that is currently absent in Sudan. Addressing this gap requires the establishment of structured institutional vaccination programs. Hospitals should provide free hepatitis B vaccines to HCWs, following the successful model implemented in Nigeria, where institutional initiatives have significantly improved vaccination rates [8].

Raising awareness through targeted education campaigns is also essential. These campaigns should prioritize high-risk groups, such as nurses, hospital cleaners, and older HCWs, who demonstrated lower vaccine uptake in our study. Educational interventions must emphasize the importance of booster doses and antibody testing to ensure sustained immunity.

Post-vaccination monitoring protocols should also be strengthened by introducing mandatory antibody titer testing and booster dose administration, aligning with international immunization guidelines [10]. Implementing these measures would facilitate the early identification of individuals with inadequate immunity and allow for timely interventions. Another crucial consideration is the development of conflict-sensitive interventions. The ongoing attacks on healthcare facilities in Sudan have significantly disrupted routine immunization services [13]. To mitigate this challenge, partnerships with non-governmental organizations should be established to ensure the continuous availability of hepatitis B vaccines and post-vaccination monitoring in conflict-affected regions. These collaborations would help sustain vaccination efforts despite healthcare system disruptions.

Recommendations

Khartoum Teaching Hospital should implement free, on-site hepatitis B vaccination drives, leveraging partnerships with the Sudan Ministry of Health and international non-governmental organizations. Expanding access to vaccination within healthcare institutions would ensure that all HCWs receive adequate protection against hepatitis B. It is also essential to mandate routine antibody titer testing and booster dose administration for HCWs, particularly those in high-risk departments. Establishing these protocols would improve immunity surveillance and enhance protection, minimizing the risk of HBV transmission within healthcare settings.

Additionally, comprehensive occupational safety training programs should be introduced to reduce the risk of infection. Needlestick injury prevention strategies must be integrated with well-defined post-exposure prophylaxis protocols, ensuring that HCWs are adequately protected from potential HBV exposure.

Limitations

This study provides critical insights into HBV vaccination gaps among Sudanese HCWs; however, several limitations must be acknowledged. First, the relatively small sample size (n=73) and single-center design restrict the generalizability of findings to broader populations or regions. Additionally, while the sample size calculation was based on established methods, the absolute margin of error was relatively high given the proportion of vaccinated HCWs, which could affect the precision of the estimates. Future studies with larger sample sizes are needed to improve statistical power and ensure more robust conclusions. Second, reliance on self-reported vaccination status introduces potential recall bias, particularly for HCWs who may have received doses in prior roles or external settings, which were not systematically verified. Third, the cross-sectional design precludes causal inferences; for instance, while needlestick injuries were associated with vaccination completion, the temporal sequence (whether vaccination preceded or followed exposure) remains unclear. Additionally, the study did not assess sociodemographic predictors (e.g., marital status, educational attainment) or HBV-specific knowledge and perceptions, which may influence vaccination behavior. Future research should prioritize longitudinal designs, verified immunization records, and inclusion of sociocultural determinants to strengthen causal interpretation and policy relevance.

## Conclusions

This study highlights critical gaps in hepatitis B vaccination coverage among HCWs at Khartoum Teaching Hospital, revealing significant barriers to complete immunization. The findings emphasize that vaccine unavailability remains the primary obstacle, compounded by financial constraints and limited awareness of the disease’s severity. While full vaccination was associated with reduced occupational exposure risk, a substantial proportion of HCWs remained unprotected, increasing their vulnerability to HBV transmission.

To mitigate these risks, urgent policy interventions are required. Institutional vaccination programs should be implemented to ensure free and readily available HBV vaccines for all HCWs. Additionally, structured post-vaccination monitoring - through routine antibody titer testing and booster dose administration - must be prioritized to enhance long-term immunity. Occupational safety measures, including comprehensive needlestick injury prevention training, should be reinforced to minimize exposure risks.
